# Task-specific CNN size reduction through content-specific pruning

**DOI:** 10.3389/frobt.2025.1552068

**Published:** 2025-09-11

**Authors:** Nurbek Konyrbaev, Martin Lukac, Sabit Ibadulla, Askhat Diveev, Elena Sofronova, Asem Galymzhankyzy

**Affiliations:** 1 Department of Computer Science, Institute of Engineering and Technology, Korkyt Ata Kyzylorda University, Kyzylorda, Kazakhstan; 2 Department of Computer and Network Engineering, Graduate School of Information Sciences, Hiroshima City University, Hiroshima, Japan; 3 Federal Research Center Computer Science and Control of the Russian Academy of Sciences (FSI), Moscow, Russia; 4 Applied Informatics and Intelligent Systems in Human Sciences Department, RUDN University, Moscow, Russia

**Keywords:** machine learning, computer vision, image classification, neural network pruning, noisy data

## Abstract

The widespread and growing use of flying unmanned aerial vehicles (UAVs) is attributed to their high spatial mobility, autonomous control, and lower cost compared to usual manned flying vehicles. Applications, such as surveying, searching, or scanning the environment with application-specific sensors, have made extensive use of UAVs in fields like agriculture, geography, forestry, and biology. However, due to the large number of applications and types of UAVs, limited power has to be taken into account when designing task-specific software for a target UAV. In particular, the power constraints of smaller UAVs will generally necessitate reducing power consumption by limiting functionality, decreasing their movement radius, or increasing their level of autonomy. Reducing the overhead of control and decision-making software onboard is one approach to increasing the autonomy of UAVs. Specifically, we can make the onboard control software more efficient and focused on specific tasks, which means it will need less computing power than a general-purpose algorithm. In this work, we focus on reducing the size of the computer vision object classification algorithm. We define different tasks by specifying which objects the UAV must recognize, and we construct a convolutional neural network (CNN) for each specific classification. However, rather than creating a custom CNN that requires its dataset, we begin with a pre-trained general-purpose classifier. We then choose specific groups of objects to recognize, and by using response-based pruning (RBP), we simplify the general-purpose CNN to fit our specific needs. We evaluate the pruned models in various scenarios. The results indicate that the evaluated task-specific pruning can reduce the size of the neural model and increase the accuracy of the classification tasks. For small UAVs intended for tasks with reduced visual content, the proposed method solves both the size reduction and individual model training problems.

## 1 Introduction

In many areas, the rapid growth of drone use and applications directly correlates with an increase in onboard processing power. This is due to the increased autonomy it provides. However, due to increasing demands for various unmanned aerial vehicle (UAV) tasks and power consumption (Abeywickrama et al., 2018; Jin et al., 2023), many of the processes required for autonomous flight and mission success must be performed off-board. For instance, advanced computer vision, reasoning, flight control, and unexpected situation-solving can be performed off-board on a powerful computer or in the cloud, and control signals can be transmitted to the UAV. This means that the UAV collects, compresses, and sends data to the operator, who makes the decision. Although this is the most natural approach, it requires reliable real-time updates from the drone sensors to the controller. This need for reliable and stable transmission can place significant stress on power-saving measures (Pinheiro et al., 2019) since the transmitter’s power is limited and signals can be lost. Automation of many drone functions can reduce power consumption, provided the task operates at a power level lower than that required for data transmission to off-board processing. For instance, small drones could execute simple computer vision tasks, such as object classification, onboard. However, the hardware limitations of onboard processors often necessitate the reduction, optimization, and adaptation of most onboard tasks. The capacity of the different hardware platforms varies depending on the UAV model, its power supply, and its intended use. Generally, as the desired functionality becomes more complex, we need to optimize the algorithms more or use a more powerful onboard hardware platform. A comparison was made by Pinheiro et al. (2019), where the authors compared off- and onboard processing for path planning. They concluded that, eventually, onboard processing is required due to transmission problems and delays. Therefore, algorithm compression is essential for compatibility with the onboard hardware and for increasing the autonomy of individual UAVs.

Currently, a large number of methods for input–output processing, decision-making, and UAV control are performed using artificial neural networks (ANNs), such as convolutional neural networks (CNNs), for instance. There are several advantages to using the neural-based approach in certain processing parts of the UAV. For instance, CNNs are highly training-dependent, learning-based tools that excel at handling noisy data. This advantage is especially important when considering that UAVs must make decisions in very noisy real-world environments. CNNs, which have been highly successful at separating noise from the content (Goodfellow et al., 2016), appear to be an efficient method when sufficient training data are available. As a result, in the past few years, UAVs and related fields have increasingly used CNNs for vision processing due to their exceptional ability to extract information from real-world data. Some examples of work include processing UVA-captured images, such as Othman and Aydin (2023), Meng et al. (2023), Osco et al. (2020), and Wijnker et al. (2021).

When it comes to optimization, ANNs have been studied to find new methods for reducing their size because large models can have billions of parameters (Brown et al., 2020). A lot of work has been carried out to minimize, reduce, and improve neural networks (Suzuki et al., 2018; Iandola et al., 2016a; Howard et al., 2017; Shimoda et al., 2017; Abdiyeva et al., 2020). Size reduction techniques have also been used to make the reduced algorithm perform better for certain tasks or the representation less sparse in general (Abdiyeva et al., 2021a). For our purposesr-reducing ANNs size for UAV hardware and tasks-we can divide size reduction and optimization of ANNs, in general, into two distinct approaches.

We refer to the first approach as the hardware-for-autonomy approach. The purpose of this approach is to reduce or optimize a given ANN/CNN to specific hardware requirements or limitations. Consequently, these methods employ various low-power hardware schemes to reduce overall power consumption. The reduction is achieved by either creating new hardware platforms that lower the total amount of power used (Li et al., 2022; Xu et al., 2021; Lamberti et al., 2022) or by suggesting operation templates with lower power consumption (Czachórski et al., 2022). Additionally, a broader research area dedicates itself to UAV computation. For instance, Paredes-Vallés et al. (2024) developed a fully neuromorphic chip for an onboard drone. Mohan et al. (2021) recently proposed a mountable framework using Jetson Nano and observed a ≈9% decrease in flight time with onboard AI processing. Rad et al. (2021) tested Jetson Nano and observed no decrease in accuracy when using CNNs for object detection. Finally, although it is not strictly related to hardware but rather to programming under constraints, a Low Power Computer Vision Challenge has been held annually since 2015 (Chen et al., 2024).

The second approach aims to reduce the code’s cost, size, or other attributes within a specific hardware framework or limits. These approaches include algorithm optimization, protocol optimization, and reducing the size of control messages. For instance, creating onboard CNNs for various visual tasks involves significant effort in training compact and energy-efficient neural networks (Othman and Aydin, 2023; Silva et al., 2020; Albanese et al., 2022). Some other projects focus on processing UAV-collected data (Meng et al., 2023; Wijnker et al., 2021) or building small, portable networks to detect drones by separating their unique sounds from background noise (Aydın and Kızılay, 2022).

The methods for the size reduction of ANNs/CNNs can, in turn, be separated into compression (quantization), precision reduction, and structural pruning.

Quantization refers to methods, like those described by Bagherinezhad et al. (2016) and Abdiyeva et al. (2018), that reduce the size of the network by storing the network parameters less accurately. For instance, Abdiyeva et al. (2018) stored all the weights in a look-up table quantized into bins. The weight retrieval process returns approximate values.

Precision reduction removes, merges, or changes CNN filters (Goyal et al., 2021; Courbariaux and Bengio, 2016. Courbariaux and Bengio (2016) binarized the network’s weights and activation functions while maintaining reasonable accuracy. Goyal et al. (2021) showed that fixed-point weight representation is possible with almost no loss of accuracy.

Finally, pruning represents a set of methods that remove certain filters or neurons from the network (Goodfellow et al., 2016). There are many approaches to removing parts of the network to reduce its size while preserving as much of the original performance as possible (Iandola et al., 2016a; Li et al., 2016; Anwar et al., 2015). For example, Iandola et al. (2016a) removed certain filters basedon their values, while Abdiyeva et al. (2021b) removed filters based on their average activation values. An intriguing result was that the authors showed that after pruning a CNN trained for object recognition, the recognition accuracy of certain classes decreased, while for others, it increased.

In this work, we expand on this finding in the context of size reduction and task specialization. We study how to reduce the size of CNNs by focusing on tailoring a neural model to UAV-specific visual tasks.

We consider the following schema. Let $D=\left\{e_{1},\ldots ,e_{n}\right\}$ be a set of UAVs and $T=\left\{t_{1},\ldots ,t_{k}\right\}$ be a set of tasks. The system assigns each task to one or multiple drones in real time, taking into account the environment and user-entered commands. For our purpose, let the task t∈T be defined as a set of required functionalities, such as object avoidance, flight stabilization, object recognition, and lion tracking. The list of functionalities specifies which software to load. Considering all the tasks that can be performed with T , a neural model that covers all of them might be too large to fit on a Jetson or a similar low-power accelerator. So, for a rapid, on-demand deployment, we investigate task-specific CNN size reduction. Specifically, we examine the vision component of an unmanned aerial vehicle (UAV) in relation to a given task (t). We consider the hardware limitations of smaller UAVs, where the available resources cannot support a CNN capable of recognizing or classifying all possible visual targets. This implies that the UAV’s GPU cannot store larger networks in real time, and smaller, function-specific networks must be used.

We consider image classification of objects obtained from an onboard camera. Each classification target is considered a task t . For the task-specific size reduction, we examine how pruning can be used to make specialized, smaller CNNs that are better at a certain visual task t . The method starts by pruning a larger network containing all the visual tasks. Using response-based pruning (Abdiyeva et al., 2021b) for a given task t , we prune the network to identify the objects. We identify the unprunable parts of the network and apply response-based pruning (RBP). RBP allows one to selectively prune the network in visual tasks (Abdiyeva et al., 2021b). The resulting pruned network is generally smaller in size and is specifically modified for task t . We evaluate the result as a difference in accuracy and provide the results for how the pruning parameters affect the resulting size and target accuracy.

To evaluate our methodology, we use the VisDrone dataset (Zhu et al., 2021). This dataset offers several tasks specifically designed for drone performance. Our research focuses on object detection and the single-object tracking challenge. For each of the datasets, we will use a pre-trained model and prune it using RBP. We directly evaluate the pruned model under various pruning parameters and compare its accuracy with the baseline accuracy offered by other state-of-the-art models. Note that we do not provide a comparative evaluation of other methodologies because, to the bestof the author’s knowledge, no other study has applied CNN pruning for context-specific size reduction. Generally, researchers use pruning as a general size reduction approach without studying an object- or class-specific size reduction. Therefore, we compare various levels of pruning as a more objective evaluation.

A summary of this work’s primary contributions:

- We evaluate a task-specific pruning methodology on a UAV-specific dataset.
- We determine the feasibility of using pruning to improve the classification accuracy of objects in UAV-related datasets.
- We demonstrate the possibility of specializing CNNs derived from large models for smaller subtasks.

## 2 Previous work

The use of CNNs or ANNs in UAVs and related applications is extensive. Researchers have explored applications, optimization, size reduction, and cost optimization in various ways and for different purposes. In this study, we mainly overview three different sets of works related to UAVs. First, we outline the applications for which ANNs are being used in UAVs; second, we outline the purpose of off-board and onboard task development; and finally, we also outline the optimization of neural networks for UAV-related tasks.

Recently, the integration of image processing and computer vision with neural networks has enhanced the capabilities of UAVs significantly. For instance, Choi et al. (2021) used a neural approach for path planning in stair climbing, while Li et al. (2023) used a neural networkfor path planning in UAV highway inspection. Direct image processing was performed by specialized CNN models, such as in Xiao (2023), Dai et al. (2020), and Wang et al. (2018). For instance, Othman and Aydin (2023) developed a low-power-specific CNN for human behavior recognition. Meng et al. (2023) developed a specific CNN for detecting small objects from the VisDrone dataset by optimizing the YOLOv7 architecture. In addition to vision, flight control has been examined in some situations, and path planning for UAVs has been researched using neural models. In the same way, CNNs have been used to identify special UAV states that enable more reliable and autonomous flight (Yang et al., 2023).

A large portion of the use of CNNs for UAVs is focused on off-board processing with the primary goal of achieving the target problem (Osco et al., 2020; Wijnker et al., 2021). More recently, several reduced-size CNNs have been developed, such as TinyNet (Dong et al., 2022), MobileNet (Howard et al., 2017), and SqueezeNet (Iandola et al., 2016b), allowing the same results to be evaluated under the hardware limitations of either platform, such as Jetson Nano TX2 or FPGA accelerators (Xu et al., 2021).

Finally, the general area of optimization includes a large amount of work that focuses on either encoding reduction (compression) or pruning (size reduction). Early examples of compression are, for instance, BinaryConnect, where the authors introduced a CNN with binary weights, or the XORNet (Rastegari et al., 2016). More recently, these reduced networks have been implemented on FPGAs (Shimoda et al., 2017) for power reduction. Abdiyeva et al. (2020) investigated a full binary CNN for general-purpose image processing. The pruning approach focuses on structurally reducing the size of the network by removing some of its components. Pruning has been viewed as a general methodto reduce the size of CNNs (Anwar et al., 2015) or make processing less expensive (Ma et al., 2019).

However, the overall performance of the network is the primary focus of all optimization methods. It is not known to the authors whether pruning has been used in a task-specific manner for improving the task’s efficiency and result accuracy.

## 3 Methodology

Generally, the need for CNN reduction stems from hardware limitations like Jetson Nano TX2, an embedded GPU accelerator. However, in this work, we emphasize that optimizing a control model for a specific context also reduces the size of the computational model. Therefore, we can view the proposed method as a second stage of a pipeline, where we first optimize a given model for the hardware and then further optimize the same model for a specific task.

**FIGURE 1:**
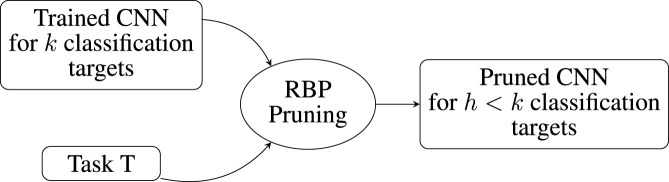
Schematic representation of the methodology for reducing a pre-trained CNN to a task-specific, smaller model.

The proposed method uses a pruning approach to specifically reduce a CNN for a target classification. The overall schematic of the proposed method is shown in Figure 1.

The method starts by taking a pre-trained CNN or training a new CNN from scratch. This CNN represents the space of all possible classification tasks T . Then, we select a specific task $t_{i}\in T$ , and we apply RBP to prune the CNN for the task $t_{i}$ . The resulting CNN is smaller and is specifically reduced to the recognition of h classification targets. The pruned network is evaluated for the classification of all k classes to determine the reliability and stability of the proposed method.

### 3.1 RBP

The reduction in the size of the control networks is studied in this section using RBP (Abdiyeva et al., 2021a; Lukac and Abdiyeva, 2023). RBP is a method of pruning neural networks.

Let $F=\left\{F_{1}\;\ldots ,F_{t}\right\}$ be the set of all filters in a CNN and $F_{i}\in R^{M\times M\times d}$ be a filter, where M is the filter’s spatial dimension and d is the depth.

Furthermore, let a subset of filters for a given CNN layer l be denoted as L such that L⊆F . Let $B\in R^{X\times Y\times d}$ be an input tensor to l ( X and Y are spatial dimensions).

To calculate the output tensor $B^{\prime }$ of the layer l , the input tensor B is convolved with the set of filters $L=\left\{F_{1}\;\ldots ,F_{d^{\prime }}\right\}$ . The output of the convolution between the input tensor B and a single filter $F_{i}$ is $R_{i}\in R^{X^{\prime }\times Y^{\prime }}$ (later referred to as a feature map), which is given by Equation 1.

$$R_{i}=F_{i}⊙B\text{, and }\;B_{\left(:,:,i\right)}^{\prime }=R_{i},$$ (1)

where ⊙ represents a convolution operation and $R_{i}$ is the component of the output tensor $B^{\prime }$ at depth i .

Let D be a dataset containing N data samples and $C=\left\{c_{1},\ldots ,c_{m}\right\}$ be the set of all object classes in the dataset. Let $D_{c}=\left\{I_{1}^{c}\ldots I_{N^{\prime }}^{c}\right\}$ be a subset of D $\left(D_{c}\subset D\right)$ containing all data samples $\left(I_{j}^{c}\right)$ with the class label c∈C . The number of samples in $D_{c}$ is denoted as $N^{\prime }$ , such that $N^{\prime }<N$ .

Finally, we denote by $R_{i}\left(I_{j}^{c}\right)$ the feature map for the filter $F_{i}$ , and the input data sample $I_{j}^{c}\in D_{c}$ .

Accumulated response ( $r_{i}^{c}$ ) for a class c and a filter $F_{i}$ is defined as the average sum of all elements in the feature map $R_{i}$ for all samples in $D_{c}$ . It is calculated as shown in Equation 2.

$$r_{i}^{c}=\frac{1}{X^{\prime }Y^{\prime }N^{\prime }}\overset{N^{\prime }}{\underset{j=1}{\sum }}\overset{X^{\prime }}{\underset{x=1}{\sum }}\overset{Y^{\prime }}{\underset{y=1}{\sum }}R_{x,y,i}\left(I_{j}^{c}\right),$$ (2)

where $R_{x,y,i}$ is a single element at a position x,y $\left(x\in X^{\prime },y\in Y^{\prime }\right)$ in a feature map $R_{i}$ .

Let $r^{c}=\left\{r_{1}^{c},r_{2}^{c},\ldots ,r_{t}^{c}\right\}$ be a vector of accumulated responses of all filters in the network for a class c . For a given pruning ratio θ∈[0,1] , RBP refers to the process of removing $|r^{c}|*\theta$ number of filters from F with the smallest $r_{i}^{c}$ values in $r^{c}$ , where $\left|r^{c}\right|$ is the cardinality of $r^{c}$ . In general, we will refer to RBP applied to a network as a pruning method $\sigma_{i}$ . For instance, for θ=0.1 , 10% of the filters with the lowest accumulated responses will be removed.

### 3.2 Network evaluation

In practice, RBP is implemented by accumulating the filter responses for a whole class of objects. This is obtained by propagating the dataset $D_{c}$ through the network, collecting the accumulated response of each filter for each input sample, and then averaging over the number of class samples to obtain $r_{i}^{c}$ . The accumulated responses are then ordered in descending order of their magnitudes, and during the pruning process, $\theta *|r^{c}|$ of them are removed starting with $r_{i}^{c}$ with the lowest values. The result of pruning $\theta *|r^{c}|$ filters is represented as a binary mask, with 1 representing filter coefficients that are not pruned and 0 representing pruned filter coefficients. This mask is the same size as the tensor representing all filters in the network. The binary mask is directly multiplied with the network filters, causing the pruned filters’ coefficients turn to 0 and thus making the filters’ output 0.

The accuracy of a network N is measured by the sum of the average class-wise accuracies, as shown in Equation 3.

$$A\left(N,D\right)=\underset{c\in C}{\sum }A\left(N,D_{c}\right)=\frac{\sum_{I\in D_{c}}\delta \left(N\left(I\right),c\right)}{\left|D_{c}\right|},$$ (3)

where D and $D_{c}$ are the whole dataset and the subset $D_{c}<D$ , respectively.

To quantify the changes in the average accuracy of the evaluated models, we define the accuracy difference ΔA such that given two networks $N_{1}$ and $N_{2}$ , we have

$$\Delta A=A\left.\left(N_{1},D\right)\right)-A\left(N_{2},D\right).$$ (4)

Note that in Equation 4, one can change the dataset to, for instance, $D_{c}$ , and the accuracy difference will show a single-class accuracy comparison between two networks. In such a case, we will use $\Delta A_{c}$ to indicate that the average accuracy is for a specific class c .

The reduction in the CNN size impacts the accuracy of the target task (Molchanov et al., 2016). For instance, in the case of visual classification, the average accuracy for a n -label classification decreases as a function of the amount of pruned cells (Molchanov et al., 2016). However, this decrease in average accuracy is not monotonic, as shown in Equation 5.

$$\begin{array}{ll} & \text{ if }\mathrm{sign}\left(\Delta A\right)<0\\ & \;\text{ then }\exists c\in C:\mathrm{sign}\left(\Delta A_{c}\right)>0. \end{array}$$ (5)

This means that when pruning a CNN in a multi-classification or recognition environment, making the network smaller can improve the accuracy of certain classes while ignoring other classes.

From a practical point of view, this means that one needs to determine whether the pruning of the already minimized networks can be used as a method of algorithm selection using a per-task configuration by pruning.

## 4 Experimental settings

### 4.1 Dataset

In this work, we use the following datasets: VisDrone (Zhu et al., 2021). The dataset contains five different tasks, namely, object detection in images, object detection in videos, single-object tracking, multi-object tracking, and, finally, crowd counting. The VisDrone dataset provides various tasks for up to 10 different object classes. Specifically, detection and tracking can be performed for the class labels and their corresponding class IDs, as shown in Table 1.

**TABLE 1:** Dataset description showing the class name, class id and the number of samples in each class.

Class name	Class ID	Class samples
Pedestrian	(1)	79,335
People	(2)	27,059
Bicycle	(3)	10,477
Car	(4)	144,847
Van	(5)	24,939
Truck	(6)	12,870
Tricycle	(7)	4,803
Awning-tricycle	(8)	3,243
Bus	(9)	5,926
Motor	(10)	29,642

The first column of Table 1 shows the class name, the second column shows the class ID, and the last column shows the number of data samples available for each class.

As observed, the training dataset is unbalanced. Furthermore, the dataset incorporates a class label for ignored regions, facilitating the training of detectors to avoid specific regions.

In the object classification task, we use all the |L|=10 labeled objects that should be identified and localized by a bounding box. To apply RBP more directly to the trained model, we reduce the detection task to the classification task. To accomplish this, we follow these steps:

1. For each sample image $I_{m}\in D$ in the dataset D that contains j objects represented by the set $O_{m}=\left\{o_{1},\ldots ,o_{j}\right\}$ , we extract each of the labeled objects $o_{k}\in O_{i}$ as image $i^{k}$ , which result in a set of images $I_{m}^{o}=\left\{i_{1},\ldots ,i_{j}\right\}$ .
2. Each object $i_{h}\in I_{m}^{o}$ is extracted to the size of the bounding box and thus is resized to the same size x×y=256×256 pixels.
3. The new dataset $D_{C}$ is the union of all images from all the sets $I_{m}^{o}$ .
4. The image set $D_{C}$ is directly associated with a set of labels $O_{C}$ created similarly by the union of all label sets $O_{m}$ .

### 4.2 Network models

We evaluate three types of convolutional neural networks. The first CNN is the simplest one. It contains only three convolutional layers, each followed by a batch normalization layer. We refer to this CNN as ${\text{CNN}}_{1}$ . The second CNN model is a set of four convolutional layers, each followed by a batch normalization layer. We refer to this CNN as ${\text{CNN}}_{2}$ . The last CNN is very similar to the first one, but each layer of convolutional filters is larger, and it is expected that this network will provide better classification accuracy. We refer to this CNN as ${\text{CNN}}_{3}$ . Each of the convolutional network contains two fully connected layers that serve as the classifier of the convoultional features to the target labels. The details of the implementation of each of the models are shown in Table 2.

**TABLE 2:** Configuration of various tested CNN models.

Network	CNN	Batch	Fully	Activation	Comments
${CNN}_{1}$	1: (10,5), 2: (20,5), 3: (20,3)	2	2	ReLU	Used dropout (0.25) for learning
${CNN}_{2}$	1: (10,5), 2: (20,5), 3–4: (20,3)	3	2	ReLU	Used dropout (0.25) for learning
${CNN}_{3}$	1: (10,5), 2: (20,5), 3–6: (20,3)	4	2	ReLU	Used dropout (0.25) for learning

The first column in Table 2 shows the name of the model. The second column shows the configuration of the convolutional layers in i:(j,k) format, where the layer ID, number of filters, and size of the filters are represented by i , j , and k , respectively. Columns three, four, and five show the number of batch normalization, fully connected layers, and the activation function, respectively. Finally, the last column describes whether any specific methods were used for learning, such as dropout, and whether other layers, such as MaxPooling, were used.

**FIGURE 2:**
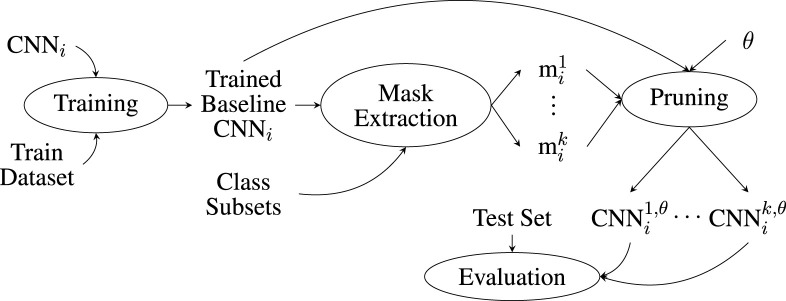
Schematic representation of the experimental steps when applying the RBP methodology used in this paper.

The experiments are conducted according to the schematic shown in Figure 2. For each ${\text{CNN}}_{i}$ , where i∈[1,2,3] , we first apply training using the training dataset. ${\text{CNN}}_{i}$ is trained to the highest possible accuracy for the classification of all |L|=k class labels in the dataset. The training parameters for all models are as follows: training epochs = 20, learning rate λ=0.001 , batch size = 256, and Adam optimizer. In addition, as shown in Table 2, dropout at a rate of 0.25 was applied during training. Each resulting baseline network ${\text{CNN}}_{i}$ is used to generate k feature maps $r^{c}$ , c=1,…,k , one for each class label k∈L . The feature maps are then analyzed using the RBP method, and a set of pruning masks $M_{i}=\left\{m_{i}^{1},\ldots ,m_{i}^{k}\right\}$ is created. The masks are applied one at a time to the network, producing the pruned network, which is shown as ${\text{CNN}}_{i}^{l,\theta }$ . This is the ${\text{CNN}}_{i}$ network that was cut down at the θ threshold for the class label l . For each ${\text{CNN}}_{i}^{l,\theta }$ , the test set is applied. Finally, the predictions of ${\text{CNN}}_{i}^{l,\theta }$ are evaluated, and the accuracy for each class is calculated.

## 5 Experiments and results

### 5.1 Full network training

The accuracy of all three networks is shown in Table 3 as average accuracy over the set of all 10 classes.

**TABLE 3:** Results of evaluation of the model CNNs trained on (a) unbalanced and (b) balanced training data.

(a)				
Class ID	Samples	${CNN}_{1}^{U}$	${CNN}_{2}^{U}$	${CNN}_{3}^{U}$
1	79,335	0.87	0.89	0.87
2	27,059	0.11	0.13	0.15
3	10,477	0.03	0.04	0.07
4	144,847	0.94	0.93	0.93
5	24,939	0.03	0.05	0.06
6	12,870	0.21	0.21	0.26
7	4,803	0.02	0.01	0.07
8	3,243	0.00	0.00	0.00
9	5,926	0.09	0.07	0.11
10	29,642	0.32	0.38	0.34

(b)			
Class ID	${CNN}_{1}$	${CNN}_{2}$	${CNN}_{3}$
1	0.62	0.67	0.57
2	0.54	0.52	0.59
3	0.49	0.55	0.56
4	0.49	0.47	0.61
5	0.43	0.37	0.43
6	0.40	0.37	0.49
7	0.39	0.41	0.35
8	0.42	0.42	0.31
9	0.65	0.65	0.71
10	0.25	0.27	0.32

Table 3a shows the result of training the networks on the data extracted from the original dataset without any post-processing. The first column in both Tables 3a,b shows the class ID, the second column in Table 3a shows the number of training samples for the unbalanced dataset, and the three last columns in both tables show the accuracy of the evaluation dataset for each of the respective three CNN models.

In Table 3a, the obtained data are highly unbalanced, and thus, the models trained on these data are referred to as ${\text{CNN}}_{1}^{U}$ , ${\text{CNN}}_{2}^{U}$ , and ${\text{CNN}}_{3}^{U}$ . We expect that training on unbalanced data will result in the classification of certain classes being significantly more accurate than others. In the present case, all three networks learned two major classes very well (based on the number of samples), namely, pedestrian (1) and car (4). In addition, classes truck (6) and motor (19) had a significant classification accuracy. The network failed to train on the remaining classes due to insufficient data representation. However, note that the number of samples is not the only factor determining the classification accuracy because classes 2 and 5 have a relatively large number of samples in the training data, but their generalization remains very low.

Table 3b shows the same networks but trained on balanced data. The balanced dataset was obtained by oversampling all the classes to the number of samples given by the class car (4), and the total resulting number of samples is 1,448,470. The results of balanced training show much more homogeneous results with all classes having a classification accuracy larger than 0.25. It is observed that using the balanced data for training resulted in all three networks having very similar performance on average. We refer to the networks trained on the multi-class dataset as ${\text{CNN}}_{1}$ , ${\text{CNN}}_{2}$ , and ${\text{CNN}}_{3}$ .

**FIGURE 3:**
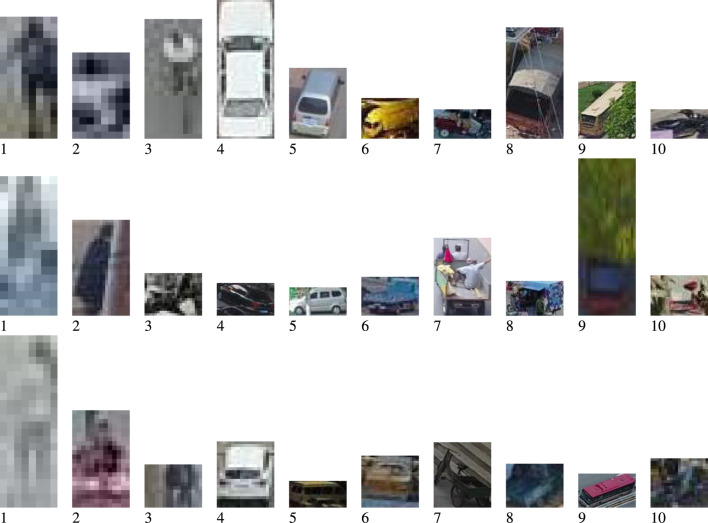
Example of images extracted from the object detection and localization VisDrone dataset.

For understanding the accuracy of the networks in Table 3, Figure 3 shows samples of each of the 10 classes of the data. It is important to note that we extracted the data specifically for object detection. We resized all the data, which can lead to significant object distortion. In addition, as shown in Figure 3, the objects are of entirely unique quality, and thus, the resulting initial performance corresponds to the dataset.

Although multi-class ${\text{CNN}}_{i}$ networks are able to learn the data, their baseline performance is not excellent.

### 5.2 Single-class network training at full accuracy

To obtain a deeper understanding of how these three networks can actually learn the classification tasks from the given dataset, we trained each of the CNNs for single object classification, one at a time. To distinguish between the multi-class trained models and the binary classifiers, we refer to the binary classifiers as ${\text{CNN}}_{1}^{s}$ , ${\text{CNN}}_{2}^{s}$ , and ${\text{CNN}}_{3}^{s}$ .

To train the binary classifiers, we created 10 different datasets (one for each target class). The size of each dataset is twice the number of samples in the target class, which is shown in the second column of Table 4. The negative label (not the target class) was randomly sampled from the remaining datasets. There were three convolutional networks trained to classify single objects. The results of their tests are shown in Table 4.

**TABLE 4:** Results of the classification using binary classifiers.

Class ID	Class name	# Samples	${CNN}_{1}^{s}$	${CNN}_{2}^{s}$	${CNN}_{3}^{s}$
1	Pedestrian	160,000	0.97	0.97	0.96
2	People	54,200	0.96	0.93	0.94
3	Bicycle	21,000	0.78	0.62	0.68
4	Car	290,000	0.20	0.20	0.05
5	Van	50,000	0.26	0.21	0.45
6	Truck	26,000	0.41	0.15	0.21
7	Tricycle	10,000	0.72	0.66	0.65
8	Awning-tricycle	6,500	0.38	0.58	0.36
9	Bus	12,000	0.33	0.23	0.35
10	Motor	60,000	0.64	0.74	0.65

**FIGURE 4:**
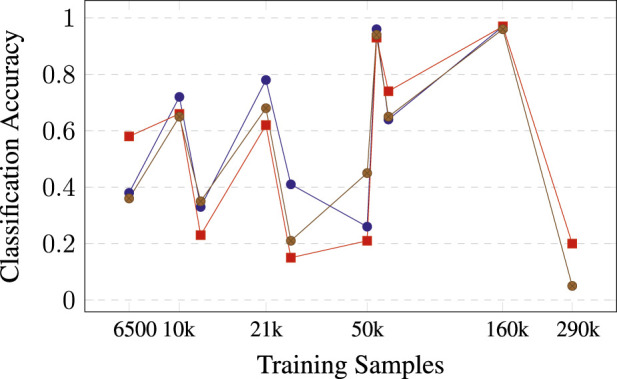
Accuracy of pruned single-class classifiers as a function of the size of the training dataset.

As anticipated, the accuracy of the binary classifiers trained for individual classes surpasses that of the networks trained for multi-class classification. This is an expected outcome as the binary classification is simpler (binary classification vs multi-class classification). In addition, for multi-class learning, the data might not be large enough. Even when oversampling is used to create a balanced dataset, the resulting dataset might not be large enough. Finally, according to the results, the network is large enough to learn a high-accuracy binary task, while for multi-class learning, a larger network could be used. For instance, assume that the verification accuracy for class pedestrian or people is over 0.95 for both classes (Table 4). This value would indicate that the network is large enough for this binary classification, but this result is not observed for all classes; thus, the size of the network is not the only parameter that would distinguish the single classifier from the multiple-class classifiers. In addition, assume that there are some outliers. In particular, Figure 4 shows the accuracy as a function of training data size. Generally, we expect classes with more samples to have higher accuracy, but when we prune a single class, dependency becomes less precise.

Furthermore, note that the multi-class CNNs are able to provide an improvement over the single-class classifiers only in specific cases. The accuracy of single-class networks is lower for certain classes compared to networks trained on unbalanced data and lower for other classes compared to networks trained on balanced data. In particular, classes truck (6), awning-tricycle (8), and bus (9) are much better recognized by the network trained on balanced data, while they are almost not recognized at all when the same network is trained on unbalanced data. The most probable reason for this result is that training it on a multi-class dataset allows it to better organize the feature space.

### 5.3 Pruning full network

#### 5.3.1 Networks trained on the balanced dataset

Pruning was performed over the set of pruning ratios specified by θ=[0.05,0.1,0.15,0.2] . We used the CNNs trained in Section 5.1 on the balanced datasets, and we pruned them for one class at a time. We then evaluated the pruned network for the classification accuracy of each of the 10 label classes.

**FIGURE 5:**
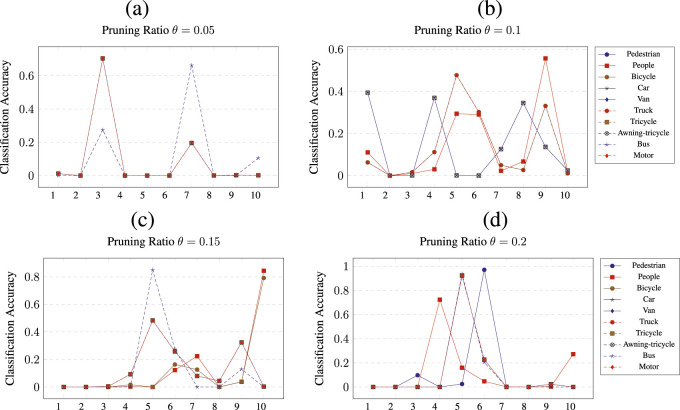
Class wise pruning of the ${\text{CNN}}_{1}$ at a) *θ* = 0.05, b) *θ* = 0.1, c) *θ* = 0.15 and d) *θ* = 0.2 pruning thresholds.

**FIGURE 6:**
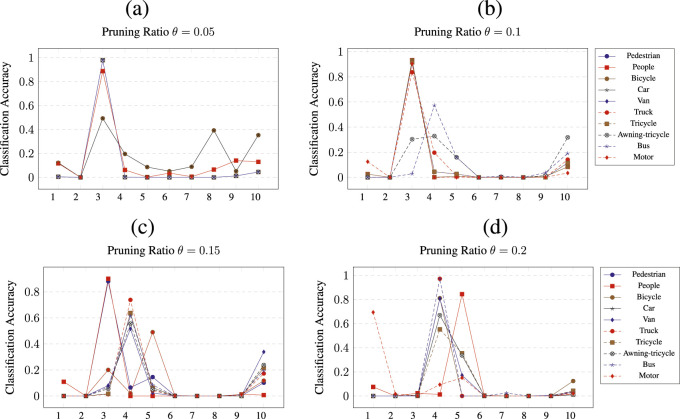
Class wise pruning of the ${\text{CNN}}_{2}$ at a) *θ* = 0.05, b) *θ* = 0.1, c) *θ* = 0.15 and d) *θ* = 0.2 pruning thresholds.

**FIGURE 7:**
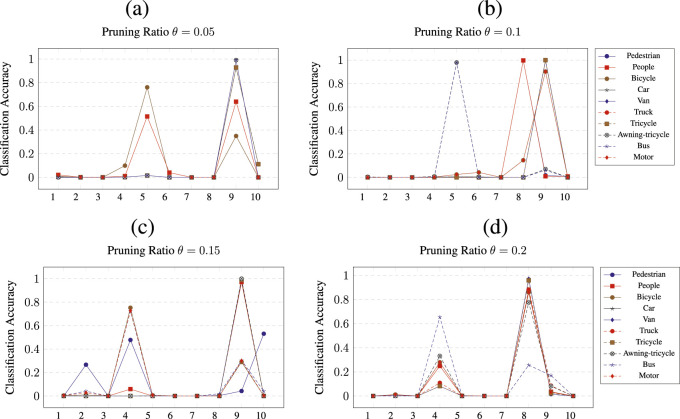
Class wise pruning of the ${\text{CNN}}_{3}$ at a) *θ* = 0.05, b) *θ* = 0.1, c) *θ* = 0.15 and d) *θ* = 0.2 pruning thresholds.

The pruning of CNNs was performed by removing θ∗100 % least active filters from the CNN. The results of pruning the three CNN networks (trained on balanced data) are shown in Figures 5–7. There are four subfigures in each figure. Each one shows the accuracy of each class when the network was pruned at the θ ratio for the respective class. In addition, each figure shows the results of the per-class pruning and the measured accuracy for all 10 classes. The x-axis is labeled by numbers from 1 to 10, representing the classes, as described in Section 4.1. Each plot contains 10 lines, each one representing a network pruned for a particular class.

Figure 5 shows the results of pruning ${\text{CNN}}_{1}$ for all four pruning ratios θ . For θ=0.05 , the network pruned for all classes except bus (9) shows a test accuracy of 70% for the bicycle class. In addition, when pruned for class bus (9), the verification for class tricycle (7) shows the classification accuracy of ≈70% . For the remaining pruned classes, the resulting network shows an accuracy of 20% or less. For a pruning ratio θ=0.1 , ${\text{CNN}}_{1}$ network displays, on average, not much improvement compared to the original unpruned network. The highest achieved accuracy is ≈60% when the network is pruned for class people (2) and is evaluated for class 9. Interestingly, when the network is pruned for classes from van (5) to bus (9), the accuracy of evaluation for classes 1, 4, and 8 increases to ≈40% . Figure 5C shows ${\text{CNN}}_{1}$ network being pruned at θ=0.15 . When pruned for class bus (9), the network shows an accuracy of 85% for class van (5), and when the network is pruned for people (1) and pedestrian (2), the network shows an improved accuracy for class 10. Finally, when pruning ${\text{CNN}}_{1}$ at θ=0.2 , the accuracy increase is the highest among all four pruning ratios. In particular, pruning for classes from bicycle (3) to motor (10) led to a classification accuracy of above 92% for classes 5 and 6.

Figure 6 shows the pruning results for all ratios θ applied to network ${\text{CNN}}_{2}$ . Here, the improvement is generally more pronounced as each threshold results in an increase in classification accuracy for at least one class, reaching 90% . For θ=0.05 , pruning ${\text{CNN}}_{2}$ for classes pedestrian (1), people (2), and motor (10) results in a classification accuracy of 88% for class bicycle (3). Pruning for classes from van (5) to bus (9) results in an improvement, with class (3) reaching 98% accuracy. For a pruning ratio of θ=0.1 , pruning only for class bicycle (3) shows an accuracy improvement of over 93% for all pruned classes except awning-tricycle (8) and bus (9). For the pruning ratio θ=0.15 , a considerable accuracy improvement is observed only when the pruned class is bicycle (3). Pruning for classes 4, 6, 7, and 9 also provides an accuracy increase above 60 % for class car (4). Finally, Figure 6D shows the pruning of ${\text{CNN}}_{2}$ at a pruning ratio of θ=0.2 . At this pruning ratio, pruning for class motor (10) results in an classification accuracy of 70% ; pruning for classes 1 and 2 results in an classification accuracy of 84% for class 4, while pruning classes truck (6) and bus (9) increases class 4 accuracy to over 95% . In addition, pruning classes bicycle (3) and van (5) increases the accuracy of class 4 to 80% .

Figure 7 shows the pruning results of ${\text{CNN}}_{3}$ . For θ=0.05 (Figure 7A), the most observable accuracy increase is for pruning of classes 4 to 9 for class bus (9) (up to 100%). A notable accuracy increase of up to ≈80% is also obtained for class van (5) when pruning for class bicycle (3). Figure 7B shows the pruning results of ${\text{CNN}}_{3}$ at θ=0.1 . For this pruning ratio, classes 5, 6, and 9 achieve an evaluation accuracy of 100% when ${\text{CNN}}_{3}$ is pruned for classes awning-tricycle (8) and bus (9), people (2), and tricycle (7), respectively. In addition, class 9 achieves an evaluation accuracy of 90% when ${\text{CNN}}_{3}$ is pruned for class truck (6). For θ=0.15 (Figure 7C), only class 9 has its accuracy improved significantly (up to 100% ) when pruning for classes people (2) and classes car (4) to awning-tricycle (8). In addition, class 4 has a classification accuracy of approximately 75% when ${\text{CNN}}_{3}$ is pruned for classes bicycle (3), bus (9), and motor (10). When ${\text{CNN}}_{3}$ is pruned at θ=0.2 , as shown in Figure 7D, class 8 is classified at an accuracy of more than 85% and up to 100% , when pruned for the classes pedestrian (1) to tricycle (7) and motor (10).

It is observed that the results show two types of patterns. First, pruning generates a complete collapse of the classification of certain classes. This result can be observed across all pruning results, such as in Figure 5A, for classes such as van (5) or bus (9). The second type of result shows that for a given pruning ratio (θ) , certain classes have their classification accuracy improved. These results can be observed in Figure 5C for classes such as van (5) or motor (10).

Next, it is observed that for the ${\text{CNN}}_{1}$ network, classes that show significantly improved classification accuracy compared to the corresponding ${\text{CNN}}_{i}^{s}$ models are van (5), truck (6), and motor (10). Other classes have had their classification accuracy increased but not by a sufficient amount to improve over the single-class classification networks. However, often, the changes in the accuracy of such classes, in general, resulted in an accuracy higher than that in the original model from Table 3b.

For network ${\text{CNN}}_{2}$ , the results of pruning are shown in Figure 6. For the classes bicycle (3), car (4), and van (5), the accuracy improved beyond that of both the single-class and multi-class CNNs.

For network ${\text{CNN}}_{3}$ , the results are shown in Figure 7. Just like the previous two pruned models, only specific classes, in this case, have benefited from pruning. In particular, these classes are van (5), awning-tricycle (8), and bus (9).

#### 5.3.2 Networks trained on the unbalanced dataset

Similar to the models trained on balanced data, we also performed network pruning on CNNs trained in Section 5.1 on the unbalanced datasets, and we pruned them for one class at a time. Pruning was again performed over the set of pruning ratios specified by θ=[0.05,0.1,0.15,0.2] , and we refer to these models as ${\text{CNN}}_{i}^{U}$ to distinguish them from the models trained on balanced data. We evaluated the pruned network for the classification accuracy of each of the 10 label classes. The purpose of pruning the unbalanced models was to observe the quantitative difference in individual class classification accuracy—improvement or reduction.

**FIGURE 8:**
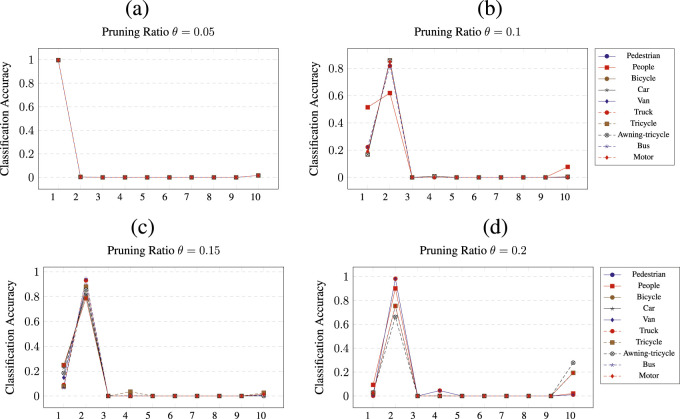
Class wise pruning of the CNN1U at a) *θ* = 0.05, b) *θ* = 0.1, c) *θ* = 0.15 and d) *θ* = 0.2 pruning thresholds.

Figure 8 shows the results of pruning ${\text{CNN}}_{1}^{U}$ . Note that for this model, only classes pedestrian (1) and people (2) have an observable improvement in classification accuracy. When the network is pruned for any of the 10 available classes, the classification accuracy of classes pedestrian (1) or people (2) is almost always increased to above 80 % . When ${\text{CNN}}_{1}^{U}$ is pruned at θ=0.05 , the classification accuracy for class pedestrian (1) reaches 100 % regardless of which class the network is pruned for. For all the other thresholds, the classification accuracy of class people (2) is improved only for most of the classes that the network is pruned for.

**FIGURE 9:**
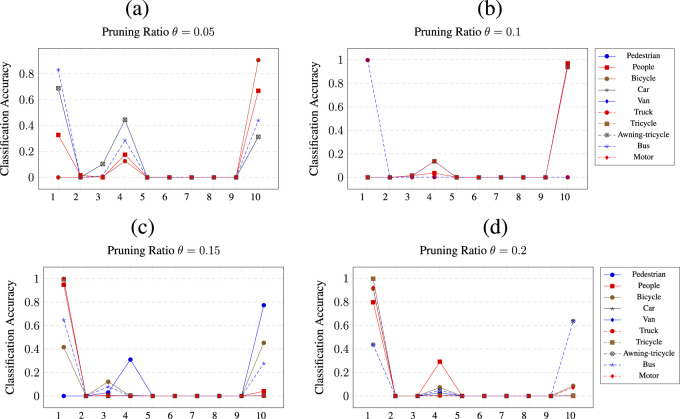
Class wise pruning of the ${\text{CNN}}_{2}^{U}$ at a) *θ* = 0.05, b) *θ* = 0.1, c) *θ* = 0.15 and d) *θ* = 0.2 pruning thresholds.

A similar result can be observed when pruning the ${\text{CNN}}_{2}^{U}$ network. The results of this pruning are shown in Figure 9. It must be observed that, again, only two classes showed improved classification accuracy across all the different pruning thresholds. When pruning ${\text{CNN}}_{2}^{U}$ at threshold θ=0.05 , the classification accuracy is improved only for classes pedestrian (1) and motor (10). However, only class motor (10) shows improvement over the classification accuracy of ${\text{CNN}}_{i}^{s}$ for motor, and that occurs when ${\text{CNN}}_{2}^{U}$ is pruned for class truck (6). For threshold θ=0.1 , the classification accuracies of classes pedestrian (1) and motor (10) are improved to up to 100 % for pruning classes truck (6) and bus (9) and all classes, respectively. For θ=0.1 , the accuracy reaches 100 % only for class pedestrian (1) when pruning tricycle (7), awning-tricycle (8), and motor (10). For threshold θ=0.2 , again only class pedestrian (1) has its classification accuracy significantly improved up to 100 % when CNN $=2^{U}$ is pruned for pedestrian (1), van (6), and tricycle (7).

**FIGURE 10:**
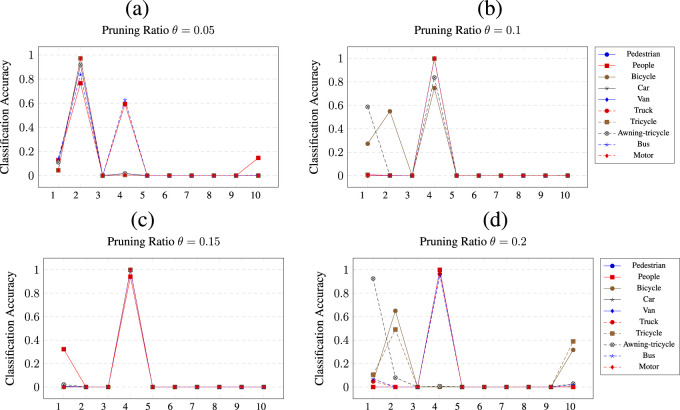
Class wise pruning of the ${\text{CNN}}_{3}^{U}$ at a) *θ* = 0.05, b) *θ* = 0.1, c) *θ* = 0.15 and d) *θ* = 0.2 pruning thresholds.

A similar picture is shown by the pruning of ${\text{CNN}}_{3}^{U}$ , as shown in Figure 10. For θ=0.05 , when pruning for truck (6) and tricycle (7), class people (2) is improved to almost 100 % classification accuracy. When pruning at θ=0.1 , class car (4) has its classification accuracy significantly improved. For pruning classes tricycle (7) and motor (10), the classification accuracy becomes 100 % for class car (4), and when pruning for class awning-tricycle, the accuracy becomes over 80 % . When pruning at a ratio of θ=0.15 , class car (4) is classified at 100 % when ${\text{CNN}}_{3}^{U}$ is pruned for any of the 10 classes. Finally, when pruning at θ=0.2 , the classification accuracy of class car (4) is significantly increased when the network is pruned for all except the tricycle (7) and awning-tricycle (8) classes.

## 6 Result discussion

By examining the evolution of class accuracy as a function of pruning and pruning masks, we can compare the results across different networks. These results are intriguing because of the following observations.

First, the three networks encode the same learned information differently. For instance, for the pruning ratio θ=0.1 , the classes that are improved by pruning are different. Table 5 shows the summary of the improved classes for the different networks and pruning ratios. Note that each network in Table 5 in the column “Network Name” represents a group of pruned networks that are pruned at a given θ . The name ${\text{CNN}}_{1}^{*,\theta }$ includes all pruned networks resulting from pruning ${\text{CNN}}_{1}^{*}$ at threshold θ for all label classes l∈[1,10] , while ${\text{CNN}}_{1}^{U,\theta }$ represents all pruned networks resulting from pruning ${\text{CNN}}_{1}^{U}$ at threshold θ for all label classes. Thus, each such group contains 10 networks.

**TABLE 5:** Summary of the classes for which the accuracy improved after RBP with respect to the original unpruned network.

Network	θ			
Group	0.05	0.1	0.15	0.2
${CNN}_{1}^{*,\theta }$	Bicycle (3)			
			Van (5)	Van (5)
			Motor (10)	
				Truck (6)
${CNN}_{1}^{U,\theta }$	Pedestrian (1)			
		People (2)	People (2)	People (2)
${CNN}_{2}^{*,\theta }$	Bicycle (3)	Bicycle (3)	Bicycle (3)	
			Car (4)	Car (4)
				Van (5)
${CNN}_{2}^{U,\theta }$	Motor (10)	Motor (10)		
		Pedestrian (1)	Pedestrian (1)	Pedestrian (1)
${CNN}_{3}^{*,\theta }$	Van (5)	Van (5)		
	Bus (9)	Bus (9)	Bus (9)	
		Awning-tricycle (8)		
				Bicycle (3)
${CNN}_{3}^{U,\theta }$	People (2)			
		Car (4)	Car (4)	Car (4)

As observed, 5 out of 10 classes were improved by pruning at various stages in networks trained on balanced data, while 4 classes showed improvement when pruning networks trained on unbalanced data. However, the representation of the information is different because not all networks prune for all five classes. No network prunes for all five classes. All networks trained on the balanced data, however, improve the bicycle and van classes. Finally, all networks trained on unbalanced data only improved classes that were represented by a larger number of training samples.

Second, not all networks ${\text{CNN}}_{i}^{*}$ improve the same classes homogeneously. For instance, Table 5 shows that ${\text{CNN}}_{2}$ improved the bicycle for three consecutive pruning ratios, but no other network improved it continuously. On the contrary, each network improved the accuracy of classes in an almost independent manner. However, no network improved more than four classes. Similarly, for the networks ${\text{CNN}}_{i}^{U}$ , the improvement can be broadly divided into two stages, one for θ=0.05 and the second for θ>0.05 ; since the networks were trained on an unbalanced dataset, pruning results in more predictable behavior.

Third, we partially achieved the original goal of distilling smaller CNNs from larger CNNs for specific subtasks. An important result here is the observation that when pruning for a specific class, the improvement is not directly predictable: in other words, pruning for a class C does not imply that the classification of class C will be increased. For instance, when pruning ${\text{CNN}}_{2}$ at θ=0.05 , class-wise pruning almost all the time improves the bicycle class. One of the reasons for this observation is that the networks are minimal, and class-wise pruning often uses the same pruning mask. Because the CNN only has so many resources, many classes exhibit similar filter responses when sorting into different groups. This implies that we can use the same filters to create the pruning masks. As a result, the pruning method cannot select class-specific filters to prune because such filters do not exist in the network. A second reason for this is that the network represents class information in proportion to the relative amount of data for each class. Therefore, pruning removes the least active filters and, as a result, tends to remove those that more strongly represent classes trained on smaller amounts of data. Consequently, the classes with the largest amount of training data will experience an increase in their classification accuracy.

Fourth, the results point to various degrees of possible overfitting. When removing filters from one class improves the classification accuracy of another class by more than 95% (such as in Figures 6A, 7B, 10), it means that the network can no longer generalize effectively over the initial set of classes. Instead, only a subset of specific filters is now working. It is intriguing to note that high accuracy was found on the evaluation dataset for both ${\text{CNN}}^{U}$ and CNN networks. The result of pruning under the experimental conditions in this paper could, therefore, be explained as non-targeted optimization: removing the set of least active filters, which are highly overlapping, results in providing a single network that improves the classification accuracy of few classes. These classes are either represented by the largest number of training samples (unbalanced training) or are classes represented most effectively by the most active features.

According to studies on class representation in CNNs (Abdiyeva et al., 2021a; Lukac and Abdiyeva, 2023), classes with similar visual features occupy nearby locations in the feature space. Pruning as a tool for removing certain features can have multiple direct effects. First, it can, as expected, remove some features and thus effectively destroy the network’s ability to recognize certain classes. Second, removing some features frees up the feature space for other features to become more prominent. Third, removing certain features allows interactions with other features to be amplified, so even classes not related to individual features can be suddenly recognized with higher accuracy. Thus, the classes with classification accuracy increases of more than 95% may be partly due to the same filters that made the pruned class perform best. However, “infecting backgrounds” can also contribute to improved classification. An object of class A can often lead to a mistaken classification when it appears with a background of class B in visual inputs. So, a high level of classification accuracy could come from both recognizing class A features and the co-occurrence statistics that come with class B features. Together, these statistics lead to a higher probability of classification.

In practice, this could imply that the networks could be potentially compressed even more. Although we consider pruning a size reduction technique in this study, it was not implemented as such. For that, the filters would have to be totally removed from the network, which is possible and would entail the expected size reduction. Considering further development, the results of pruning and isolating filters for individual or groups of classes provide a roadmap for designing optimal object classifiers or feature extractors. In addition, understanding which filters are important to a given class and identifying the interfering background are also helpful in determining the optimal size of classifiers.

Finally, the fact that only certain classes are improved by pruning can also be observed from the point of view of pruned filters, i.e., pruned masks. Examining the overlap of the masks for different classes at different θ , we note that the average similarity between binary masks is 80% . This means that due to the small size of tested networks, the classes are represented by filters, and their distinction is induced by a very small number of low-active filters. Pruning these filters, thus, causes certain classes to become completely unrecognized, while other classes become recognized at very high accuracy.

## 7 Conclusion

In this paper, we show the application of neural network pruning as a method for generating neural networks for selective classification. The proposed method for generating specialized CNNs from a large-scale CNN showed promise for certain classes of objects. In addition, the class-wise RBP showed that, while being exclusive, it also positively affects other classes. We can see this as a side effect of the de-cluttering of the feature space where individual object classes are located.

## Data Availability

Publicly available datasets were analyzed in this study. These data can be found at: https://github.com/VisDrone/VisDrone-Dataset.
